# Mutational burden and chromosomal aneuploidy synergistically predict survival from radiotherapy in non-small cell lung cancer

**DOI:** 10.1038/s42003-021-01657-6

**Published:** 2021-01-29

**Authors:** Qingzhu Jia, Qian Chu, Anmei Zhang, Jing Yu, Fangfang Liu, Kaiyu Qian, Yu Xiao, Xue Wang, Ying Yang, Yi Zhao, Ji He, Guanghui Li, Yisong Y. Wan, Conghua Xie, Bo Zhu

**Affiliations:** 1 Department of Oncology, Xinqiao Hospital, Army Medical University, Chongqing, 400037 China; 2 Key Laboratory of Immunotherapy, Xinqiao Hospital, The Third Military Medical University, Chongqing, 400037 China; 3 Department of Oncology, Tongji Hospital, Huazhong University of Science and Technology, Wuhan, Hubei China; 4 Department of Radiation and Medical Oncology, Zhongnan Hospital of Wuhan University, Hubei Key Laboratory of Tumor Biological Behaviors, Hubei Cancer Clinical Study Center, Wuhan, 430071 Hubei China; 5 Department of Biological Repositories, Zhongnan Hospital of Wuhan University, Wuhan, China; 6 Department of Thoracic Surgery, Tongji Hospital, Huazhong University of Science and Technology, Wuhan, 430071 Hubei China; 7 GeneCast Biotechnology Co., Ltd., 35 North Haidian Rd, HealthWork Suite 901, Beijing, China; 8 Department of Microbiology and Immunology, Lineberger Comprehensive Cancer Centre, University of North Carolina at Chapel Hill, NC, USA

**Keywords:** Predictive markers, Translational immunology

## Abstract

Therapeutic radiation can result in substantially different survival outcomes for patients with non-small cell lung cancer (NSCLC). Measures for identification of patients who can benefit most throughout radiotherapy remain limited. In this retrospective study, survival analysis was performed based on a discovery cohort from TCGA and a validation cohort from three independent hospitals. Tumor mutational burden (TMB) and chromosomal aneuploidy (ANE) were derived from the whole exome sequencing (WES) data from treatment-naïve tumors. Integrated risk scores were derived from TMB and ANE by a multivariate Cox proportional hazards model. TCGA reveal that TMB and ANE are associated positively and negatively, respectively, with survival throughout radiotherapy. Additionally, the synergistically predictive significance of these two genomic alterations, in differing responders and non-responders to radiotherapy is identified. These biomarkers may have clinical potential to improve personalized treatment management by rationally identifying highly likely responders to therapeutic radiation in patients with NSCLC.

## Abstract (web summary)

Here, the authors use whole exome sequencing to determine the effect of tumor mutational burden (TMB) and chromosomal aneuploidy (ANE) on survival outcomes of non-small cell lung cancer patients undergoing radiotherapy. The authors find that a higher TMB and lower ANE were correlated with better survival outcomes and that these factors interact synergistically.

## Introduction

The efficacy of therapeutic radiation varies substantially across different individuals with non-small cell lung cancer (NSCLC). Currently, few consensus biomarkers exist to predict treatment response to radiotherapy for NSCLC. Therefore, identifying the parameters potentially associated with favorable treatment efficacy to radiotherapy is a worthy area of translational investigation.

A growing body of preclinical and clinical observations has emphasized the immunomodulatory effects of radiotherapy in anti-tumor treatments, especially noteworthy in the era of immunotherapies. Ionizing radiation has been demonstrated to elicit immunogenic cell death which can effectively activated neoantigen-specific T-cells and downstream immune response^1,2^. The PACIFIC^3,4^ trial, which first demonstrated a dramatic increase in the survival benefits for patients treated with definitive chemoradiation and subsequent immune checkpoint blockades (ICB), strongly supported the idea of an immune-provoking effect of radiotherapy in clinical practice. Moreover, data from both KEYNOTE-001^5^ and PEMBRO-RT^6^ trials demonstrated improved objective response rate and considerably prolonged the survival outcomes, by performing radiotherapy in patients with metastatic NSCLC who received subsequent pembrolizumab administration. Collectively, the issue of radiation-mediated immunomodulation could convey survival benefits bolsters the hypothesis that the efficacy of radiotherapy could be predicted through investigating the intrinsic immunogenicity of the tumor microenvironment.

Considering that the tumor mutational burden (TMB)^7^ and chromosomal aneuploidy (ANE)^8^ are now widely recognized as critical determinants of the immunogenicity^9^ of the tumor microenvironment, we investigated the significance of these parameters in predicting the efficacy of radiotherapy in patients with NSCLC. To this end, we analyzed the genomic and clinical information from The Cancer Genome Atlas (TCGA)-derived discovery cohort, and an independent validation cohort comprising NSCLC patients enrolled in three hospitals.

## Results

### Predictive value of total tumor mutational burden on survival outcomes from radiotherapy

We investigated the association between TMB, as derived from the whole-exome sequencing (WES) data in TCGA database^10^, and the survival follow-up after treatment with radiotherapy for patients with NSCLC. In the discovery cohort, 113 NSCLC patients who had undergone radiotherapy and whose tumors were profiled by both WES and RNA-sequencing (RNA-Seq) were included. The control cohort consisted of 738 patients who had not received radiotherapy (see details in Data availability). Mutation count was downloaded from the cBioportal.

**Fig. 1:**
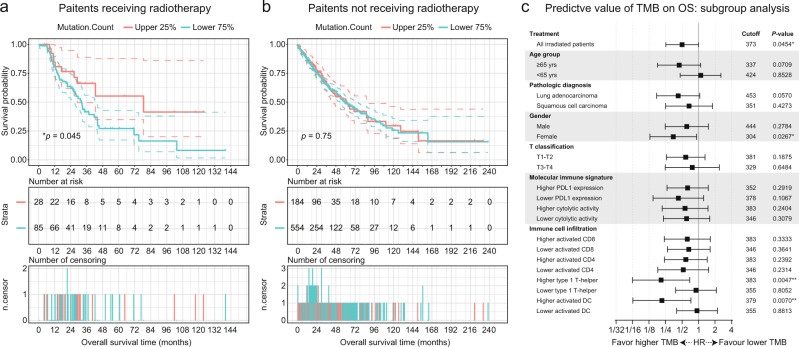
Survival according to the presence of high or low tumor mutational burden within tumor. **a, b** Kaplan-Meier survival curves of OS (upper panel), table of number of risk (middle panel), and number of censoring (lower panel) according to the different mutational burdens for patients with (**a**) or without (**b**) radiotherapies. The hazard ratio (HR) was estimated by means of Log-rank test. 95% CI for HR was shown as colored dashed line. Red line, patients with higher TMB; blue line, patients with lower TMB. **c** Forest plot of TMB on OS for radiated patients, stratified by demographics, molecular immune signatures, and immune cell infiltration. HRs comparing OS within each subgroup were showed in log2 scales. Bars represent the 95% CI. The upper 25% cutoff TMB for each subgroup as well as the two-sided Cox proportional regression *p*-value were displayed. The molecular immune signature and immune cell infiltration were grouped based on their median value.

A screening assay was performed to identify an optimized threshold for categorizing the subgroups of patients (Supplementary Fig. 1). Considering the limited number of patients, we assessed the cutoff for TMB from 20% to 80% using a 1% stepwise function to balance the representativeness and statistical power of screening assay. By calculating the hazard ratio (HR) against every cutoff, we found a general association of improved survival in the higher TMB groups (Supplementary Fig. 1a, HR < 0.8). This clinical benefit remained relatively consistent when the HRs were adjusted using sex (male vs female), age (≥65 year vs <65 year), and the pathological diagnosis (lung adenocarcinoma vs squamous cell lung cancer) for the cohort (Supplementary Fig. 1b, HR < 0.8). In summary, we observed an average 40% decreased hazard (HR ~ 0.6, ranging from ~0.4 to ~0.8) for patients with higher mutation burden, roughly corresponding to upper 25th or 75th percentile. In representative publications which investigated the predictive significance of TMB^11^, higher TMB group usually contains less than half of the cohort. Therefore, the higher TMB group was defined as the top 25% of the population (cutoff_TMB_ = 373) in our following analyses. Survival analysis indicated durable survival for the irradiated cohort with a higher TMB (Fig. 1a, *p = 0.0454; HR = 0.511; 95% confidence interval (CI), 0.2911–0.8971). In contrast, for patients who did not receive radiotherapy, carrying a higher TMB did not translate into a survival superiority (Fig. 1b, *p* = 0.7534; HR = 0.9572; 95% CI, 0.7302–1.2550), indicating that the predictive significance of TMB is limited to the subset of patients who had received radiotherapy.

Through consequent stratified analysis, an association of prolonged survival across almost all the stratifications was demonstrated (Fig. 1c and Supplementary Data 1; the immune cell infiltration was estimated by single-sample gene set enrichment analysis (ssGSEA)^12,13^). Although the effects for most stratifications did not reach statistical significance, likely due to the relatively small sample size, the numerical trends of better survival were observed in nearly all the subgroups.

### Predictive value of chromosomal aneuploidy on the survival outcomes from radiotherapy

**Fig. 2:**
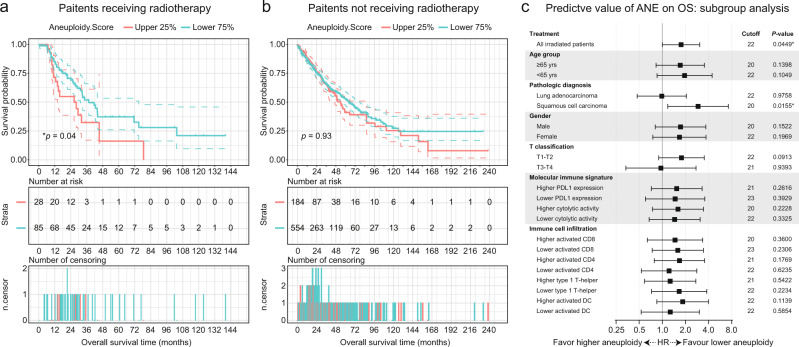
Overall survival according to the presence of high or low tumor aneuploidy score within tumor. **a, b** Kaplan-Meier survival curves of OS (upper panel), table of number of risk (middle panel), and number of censoring (lower panel) according to the different aneuploidy (ANE) scores for patients with (**a**) or without (**b**) radiotherapies. The hazard ratio (HR) was estimated by means of Log-rank test. 95% CI for HR was shown as colored dashed line. Red line, patients with higher ANE score; blue line, patients with lower ANE score. **c** Forest plot of ANE score on OS for radiated patients, stratified by demographics, molecular immune signatures, and immune cell infiltration. HRs comparing OS within each subgroup were showed in log2 scales. Bars represent the 95% CI. The upper 25% cutoff ANE score for each subgroup as well as the two-sided Cox proportional regression *p*-value were displayed. The molecular immune signature and immune cell infiltration was grouped based on their median value.

Chromosomal ANE refers to the presence of an abnormal number of chromosomes in reference to a haploid set. It was reported to correlate with the inferior clinical response for patients administered with ICBs^8^. Here, we next assessed the predictive value of ANE (downloaded from the cBioportal) on radiotherapy. The higher ANE group was defined as the top 25% of the patients with ANE (cutoff_ANE_ = 22). The lower ANE group showed significantly improved survival for patients who had received radiotherapy, suggesting ANE also influences outcomes (Fig. 2a, *p = 0.0450; HR = 1.7360; 95% CI, 0.9202–3.2770). For patients who had not received radiotherapy, we observed nearly identical survival prognosis between the different ANE groups (Fig. 2b, *p* = 0.9313; HR = 0.9879; 95% CI, 0.7498–1.3020), supporting ANE score as an adverse indicator of benefiting from radiotherapy. Stratified analyses validated the numerical trends that greater ANEs were negatively associated with the clinical benefits across most of the subgroups, except for patients with diagnoses of lung adenocarcinoma or with T3-T4 tumor staging (Fig. 2c).

### Synergistic predictions based on TMB and ANE

**Fig. 3:**
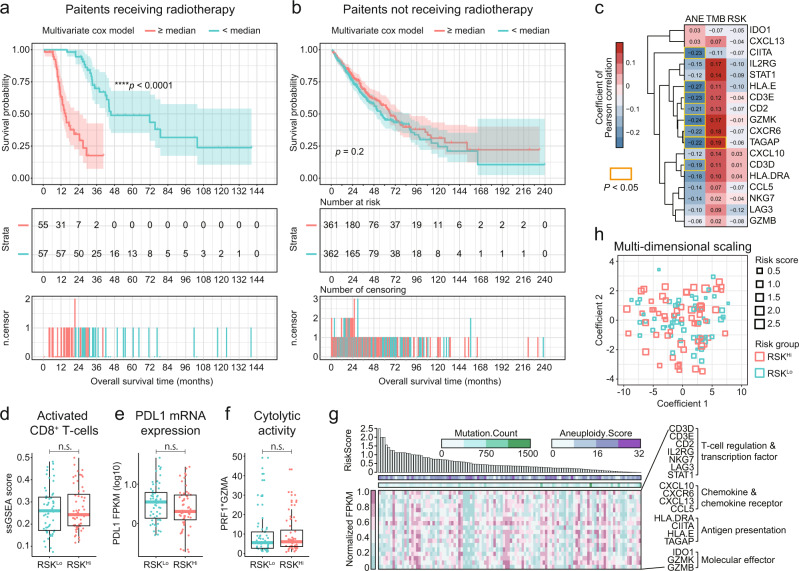
Risk score predicts survival outcome and is not related to the tumor immune microenvironment. **a, b** Kaplan-Meier survival curves of OS (upper panel), number of risk (middle panel), and number of censoring (lower panel) patients according to the different risk scores (RSKs) for patients with (**a**) or without (**b**) radiotherapy. The hazard ratio (HR) was estimated by means of log-rank test, with 95% CI for HR shown as a dashed line. The red line indicates patients with a higher RSK, while the blue line indicates patients with a lower RSK. **c** Heatmap comparison of the correlation between T-cell inflammatory signature genes and three predictive biomarkers. Pearson correlation coefficients are shown. Statistically significant correlations (*p* < 0.05) are outlined with yellow squares. The color gradient bar represents the correlation coefficient. **d**–**f** Comparison of infiltration of activated CD8^+^ T-cells (**d**), PDL1 mRNA expression (**e**), and cytolytic activity (**f**) between risk groups. Two-tailed Mann-Whitney *U*-test was used for all comparisons. Boxplot and error bars represent the median ± range. A *p* ≤ 0.05 was considered statistically significant for all comparisons. **g** Measurement of 18 genes in an IFN-γ-related GEP for each irradiated patient. Mutation count, aneuploidy score, and RSK are annotated in the upper panel. Patients were ordered by decreasing RSK. **h** Multidimensional scaling (MDS) based on T-cell inflammatory signature genes. The color and size of each square indicates the risk group and score for each patient.

Since correlation analysis showed that TMB and ANE are independent predictive factors for patients with NSCLC **(**Supplementary Fig. 2, R2 = 0.04, *p = 0.03**)**, we further evaluated whether the joint utility of these two predictive indicators could improve the predictive power in the clinical benefits from radiotherapy. To evaluate the predictive significance of TMB and ANE simultaneously, we employed a multivariate Cox proportional regression approach to derive a representative risk score (RSK) as established previously^14^. Multivariate Cox proportional regression model was employed to derive an integrated RSK for each patient^14^ (RSK = 18.2*ANE – 1.1*TMB; Supplementary Data 2), with patients categorized along the median RSK. The baseline clinical characteristics were comparable between the higher and lower RSK groups **(**Supplementary Data 3**)**. Using this prediction model, we achieved an improvement in the magnitude to HR in comparison to either individual predictive indicator (Fig. 3a, ^****^p < 0.0001; HR = 4.1684; 95% CI, 2.3047–7.5358). Furthermore, when this prediction model was applied to patients who had not undergone radiotherapy, we still observed a statistically insignificant difference in the survival of patients with distinct RSKs (Fig. 3b, *p* = 0.1961, HR = 1.1720; 95% CI, 0.9217–1.4890).

### Risk score is independent from the tumor immune microenvironment

T-cell inflamed gene expression signature associated with the survival outcomes of patients receiving immunotherapy^15^. TMB was positively correlated, while ANE was negatively correlated to the inflamed immune microenvironment and the cytolytic function of infiltrated T-cells^8^. In the present study, an IFN-γ-related gene expression panel (GEP) with 18 genes was used to characterize the tumor immune microenvironment^15^. As expected, TMB had positive associations with the GEPs, while ANE had negative associations, which was consistent with previously published evidence (Fig. 3c and Supplementary Figs. 3 and 4). Notably, in contrast to the TMB and ANE, none of the genes in the GEP were statistically significantly correlated to the RSK (highlighted by the opened yellow square, *p* < 0.05), implying that the integrated RSK was independent from the tumor immune microenvironment (Fig. 3c and Supplementary Fig. 5). We then compared the key immunological elements in cytotoxic T-cell function directly between different risk groups. Cytolytic activity, which is derived from *PRF1* and *GZMA* gene expression, was used to quantify the magnitude of the function of cytotoxic T-cells^16^. No significant differences were observed between the tumor immune microenvironments in patients of higher and lower risk groups, whether by measuring the infiltration of activated CD8^+^ T-cells (Fig. 3d), the level of PD-L1 mRNA expression (Fig. 3e), or the cytolytic activity (Fig. 3f). We further confirmed that the RSKs did not depend on the IFN-γ-related GEP and the magnitude of immune cell infiltration (Fig. 3g and Supplementary Fig. 6). When the bio-similarity between immune microenvironments was evaluated and visualized using the multidimensional scaling (MDS)^17^, the patients with distinct RSKs were distributed uniformly, demonstrating that the RSK did not rely on immune microenvironment prior to receiving radiotherapy (Fig. 3h).

### Predictive significance of RSK in validation cohort

**Fig. 4:**
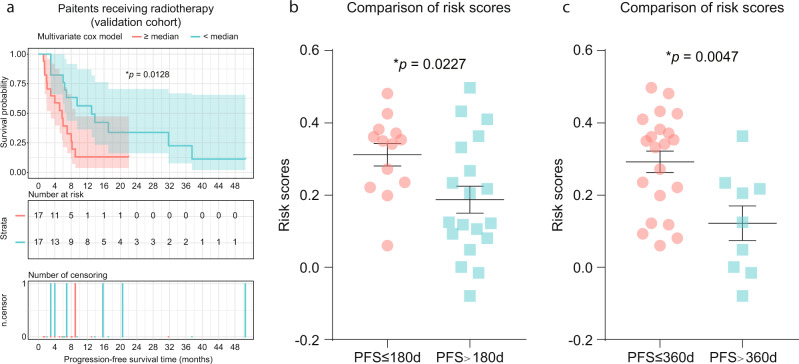
Confirmation of risk scores’ predictive power in the survival outcome for the validation cohort. **a** Progression-free survival (PFS) for patients in the validation cohort. The hazard ratio (HR) was estimated by means of a Log-rank test. The 95% CI for HR was shown as a dashed line, in the respective color. Red line, patients with a higher risk score (RSK); blue line, patients with a lower RSK. **b**, **c** RSKs between varied survival benefits from radiotherapy. PFS ≤ 180 or 360 days, patients reached endpoint earlier than specified days; PFS > 180 or 360 days, patients reached endpoint later than specified days, or ongoing response at specificed days. Horizontal line, mean value; error bars, s.e.m. Statistics based on two-tailed Student’s *t*-test.

Finally, we aimed to examine the predictive significance of the TCGA-derived RSK model in an independent validation cohort comprising 34 patients who were diagnosed and sampled at three hospitals (Supplementary Data 4). TMB and ANE were calculated based on pretreated tumor tissues using established methods (Supplementary Data 4). Comparison of the baseline between the discovery and validation cohorts is shown in Supplementary Data 5. For the predictive significance of TMB or ANE alone, both individual predictors showed consistent impact on the survival outcome for patients receiving radiotherapy (TMB, cutoff_TMB_ = 185 or 5.95/Mb, *p* = 0.2859, HR = 0.6181; ANE, cutoff_ANE_ = 22, *p* = 0.8117, HR = 1.109). However, their predictive values remain statistically insignificant in our validation cohort, probably due to the limited cohort size. In the assay of their integrated RSK, we identified significantly favorable survival outcome for patients with lower RSK (Fig. 4a, ^*^p = 0.0128, HR = 2.38, 95% CI, 1.054–5.373). Furthermore, by comparing the RSKs between patients progressed and not progressed at 180 days (Fig. 4b, *p = 0.0227) and 360 days (Fig. 4c, ^**^p = 0.0047), respectively, we observed consistently and significantly lower RSKs for patients with durable survival time, further supporting the predictive value of RSK in radiation treatment.

## Discussion

To our knowledge, this study is among the first to report that two major measurements of tumor immunogenicity, TMB and chromosomal ANE, respectively, could synergistically predict the efficacy of radiotherapy for patients diagnosed with NSCLC. Although the univariate analysis suggested that both TMB and ANE are informative for predicting the clinical outcomes with respect to radiotherapy, a multivariate Cox proportional regression model enabled us to determine the relative weight of these two independent parameters and to significantly improve the performance of prediction. Survival analysis of patients without a history of receiving radiotherapy revealed that TMB and ANE had no predictive value. The robustness of the integrated RSKs was confirmed in the publicly accessible discovery cohort, and an in-hospital validation cohort.

Somatically, genomic alterations form the genetic basis of intrinsic immunogenicity in tumor cells. Radiation induces a local inflammatory microenvironment and enhances the expression/release of *HMGB1*, which directly promotes inflammatory signaling by interacting with *TLR2*, *TLR4*, or *RAGE*^18,19^. Proper radiotherapy should theoretically overcome the suppressive tumor immune microenvironment and facilitate the recognition of radiation-exposed neoantigens. Therefore, it is reasonable to expect that TMB and ANE, two robust measurements of genomic alterations, could be the indicators for survival benefits from radiotherapy. In the present study, we did not observe different tumor immune microenvironments in patients with distinct risk stratification prior to receiving radiotherapy (Fig. 3). Considering that radiation can overwhelmingly reprogram the immune features of the tumor microenvironment, it is not a paradox that the RSK is irrelevant to the baseline tumor immune microenvironment prior to radiotherapy.

Potential limitations of the study are as follows: (1) Most of our analyses were based on biopsied tumor tissue as a whole, which lacked the resolution to investigate the spatial heterogeneity^17^ of the tumor mass; (2) Despite success with the discovery cohort, we were unable to validate the predictive significance of TMB and ANE alone in the validation cohort, probably due to the small size of this cohort (*N* = 34); however, our data suggest the relatively weaker power of TMB or ANE as individual indicators for responders to therapeutic radiation; (3) Due to the availability of biopsies, the clinical characteristics between the discovery and validation cohorts were not satisfactorily balanced; for example, the validation cohort comprised more younger patients as well as more patients with T3 or T4 disease than those of the discovery cohort. Thus, future stratified analyses are important for evaluating the predictive significance of RSK in subgroups; (4) A clinically applicable cutoff for TMB/ANE or RSK should be carefully determined in further perspective studies in a broader numerical range, and with a larger cohort size; (5) We are yet to validate whether the association between these genomic alteration measurements and response to radiotherapy was universal for all/multiple solid tumors or types specific to NSCLC.

We expect that through a powered trial-design, the synergistic role of TMB and ANE in identifying the potential responders to radiotherapy could be further validated, leading to a better understanding of the mechanisms underlying the radiation-induced tumor elimination in NSCLC. Furthermore, as informative roles, TMB and ANE could possibly provide an instructive avenue for identifying candidates influencing radio-combinational treatment modalities in multiple solid tumor types.

## Methods

### Sources of publicly accessible data, patients, and sample collection

TMB and ANE of discovery cohort were downloaded from the cBioportal database. Patients with the following criteria were enrolled into the validation cohort: (1) diagnosed with NSCLC and received radiotherapy at XinQiao Hospital (Army Medical University, Chongqing), TongJi Hospital (Huazhong University of Science and Technology, Wuhan), or Zhongnan Hospital (Wuhan University, Wuhan); (2) at least 18 year old at diagnosis; (3) availability of radiological surveillance scans; (4) at least 10 slides of 5 μm thick formalin-fixed paraffin-embedded (FFPE) biopsies and peripheral blood mononuclear cell (PBMC) from a total of 2 ml of blood that was obtained at the time of diagnosis. The study was approved by the institutional review board of XinQiao Hospital (Army Medical University, Chongqing), TongJi Hospital (Huazhong University of Science and Technology, Wuhan), and Zhongnan Hospital (Wuhan University, Wuhan). The Declaration of Helsinki was followed in this study. All patients in validation cohort provided written informed consent.

### In silico estimation of immune cell infiltration

The single-sample gene set enrichment analysis (ssGSEA)^12^ was employed to estimate the infiltration of immune cells^17^. R package “GSVA” was used to calculate the ssGSEA score. Feature gene panels for 28 immune cell types were obtained from a recent publication^20^. A ssGSEA score was calculated to represent the relative abundance of each type of immune cell.

### Multivariate Cox model and survival analysis

For the survival analysis based on single predictive indicators, we performed Log-rank tests and utilized a univariate Cox proportional regression model, using R package *survival* and visualized the results using R package *survminer*. We selected TMB and ANE as two independent predictive indicators. Patients who received or did not receive radiotherapy were grouped into higher and lower subgroups, based on their TMB/ANE in comparison to the upper 25% quantile values. The HRs and 95% CIs were also determined.

The multivariate Cox proportional regression model was used to determine the relative contribution of TMB and ANE, as predictive indicators of survival. Both predictive indicators were fitted in a Cox multivariate model. Predictive RSKs were derived from fitting the model^8^. The median RSKs were used to group the patients into high- and low-risk groups. The HR and 95% CI were determined for this RSK. In addition, the individual HR and *p*-values for the two predictive indicators in the Cox fitting model were also calculated. One irradiated without ANE score was excluded from the subsequent analysis.

### DNA extraction and sequencing of validation cohort

Samples of at least 20% tumor content were subjected to DNA extraction. DNA was extracted using BLACK PREP FFPE DNA kit-50r kits (AJ Innuscreen GmbH - Analytik Jena, Berlin, Germany) as per the manufacturer’s SOP. Samples with less than 500 ng gDNA, or gDNA degradation as confirmed via agarose gel electrophoresis detection, were excluded. After fragmentation via Covaris M220 (COVARIS Inc., USA), KAPA Hyper Prep Kit (Illumina platforms) (KAPA Biosystems, Massachusetts, USA) was used for library preparation, including end repair, 3′ end addition, and adapter ligation. Exon regions were captured using the NimbleGen SeqCap EZ Exome Library kit (Roche, Wisconsin, USA). Sequencing was done on the Illumina NovaSeq6000 platform using PE151 strategy at Genecast (Beijing) Biotechnology Co., Ltd.

### Data analysis for whole-exome sequencing of validation cohort

After mapping the qualified reads to the reference genome (hg19) using the BWA program^21^, single nucleotide variants (SNVs) and small insertions and deletions (INDELs) were called using the VarDict^22^ software, and FreeBayes software was used for complex mutation calling. Then all variants were annotated with ANNOVAR^23^. Based on the variant QC metrics and annotation results, only somatic variants that meet the following criteria were used for downstream analyses: total read depth ≥40×, VAF ≥ 5%, with supporting reads on both strands, annotated as nonsynonymous variants, and with a corresponding minor allele frequency of ≤0.002 in both the Exome Aggregation Consortum (ExAC) database^24^ and the Genome Aggregation Database (gnomAD)^25^.

### Calculation of tumor mutational burden of validation cohort

The TMB value was determined by the total number of somatic nonsynonymous variants, and the value was shown in absolute number or was normalized by the covered whole-exome base, using a validated algorithm^26^. TMB was measured in terms of the absolute number and normalized number of mutations, per million exonic bases, for patients in validation cohort.

### Calculation of aneuploidy score of validation cohort

Batch model of CNVkit (v0.9.2)^27^ was used for each set of paired samples, which generated the log2 value of the copy numbers in each exonic segment, as designated with the input BED file. After which, the log2 value of each chromosome arm was then calculated using the *segment* module with the segmentation algorithm *none*. Then copy number of each arm was calculated using the *call* module, with specified purity calculated by Sequenza (v2.1.2)^28^, the output of which was expressed as absolute integer numbers. Arms with copy number ≥3 (gain) or ≤1 (loss) were defined as “altered”. Lastly, the ANE score was defined as the total number of altered arms in the sequenced sample^29^.

### Statistics and reproducibility

Two-tailed Student’s *t*-test or Mann-Whitney *U*-test were performed for comparison of the parametric or non-parametric datasets, respectively. Survival outcomes were measured with either progression-free survival or overall survival depending on data availability of the cohort. The events were defined as progression or death due to any cause. Kaplan-Meier survival curves were generated to show the different survival rates between the groups. A *p*-value of less than 0.05 was considered significant. The predictive significance of risk score was confirmed in two independent cohort as replication.

### Reporting summary

Further information on research design is available in the Nature Research Reporting Summary linked to this article.

## Supplementary information

Supplementary information.

Description of Additional Supplementary Files.

Supplementary Data 1.

Supplementary Data 2.

Supplementary Data 3.

Supplementary Data 4.

Supplementary Data 5.

Reporting summary.

## Data Availability

For the discovery cohort, 566 patients with lung adenocarcinoma (LUAD, https://www.cbioportal.org/study/summary?id=luad_tcga_pan_can_atlas_2018) and 487 patients with lung squamous cell carcinoma (LUSC, https://www.cbioportal.org/study/summary?id=lusc_tcga_pan_can_atlas_2018) in the TCGA database were enrolled. In all, 113 patients with “YES” and 738 patients with “NO” in the item “Radiation Therapy” were enrolled as the discovery cohort for patients that had received and not received radiotherapy, respectively. The remaining 202 patients with “NA” for the item “Radiation Therapy” were excluded from subsequent analysis. For the validation cohort, original sequencing data supporting the conclusions of this project have been deposited in the Sequence Read Archive (SRA) under accession number SRP251644.

## Code availability

All analyses were based on established bioinformatics pipelines as showed in citations and described in the Methods.
