# Supplementary material for: Mutational burden and chromosomal aneuploidy synergistically predict survival from radiotherapy in non-small cell lung cancer
Source: Commun Biol. 2021 Jan 29;4:131. doi: 10.1038/s42003-021-01657-6 (PMC7846582; doi:10.1038/s42003-021-01657-6)
Supplement: Supplementary file 1 — Supplementary information. [file 42003_2021_1657_MOESM1_ESM.pdf]

Supplementary Information for

Mutational Burden and Chromosomal Aneuploidy Synergistically Predict Survival from Radiotherapy in Non-Small Cell Lung Cancer

Qingzhu Jia†, Qian Chu†, Anmei Zhang†, Jing Yu, Fangfang Liu, Kaiyu Qian, Yu Xiao, Xue Wang, Ying Yang, Yi Zhao, Ji He, Guanghui Li, Yisong Y. Wan*, Conghua Xie*, Bo Zhu*

Supplementary Figure 1-6

Supplementary Figure 1

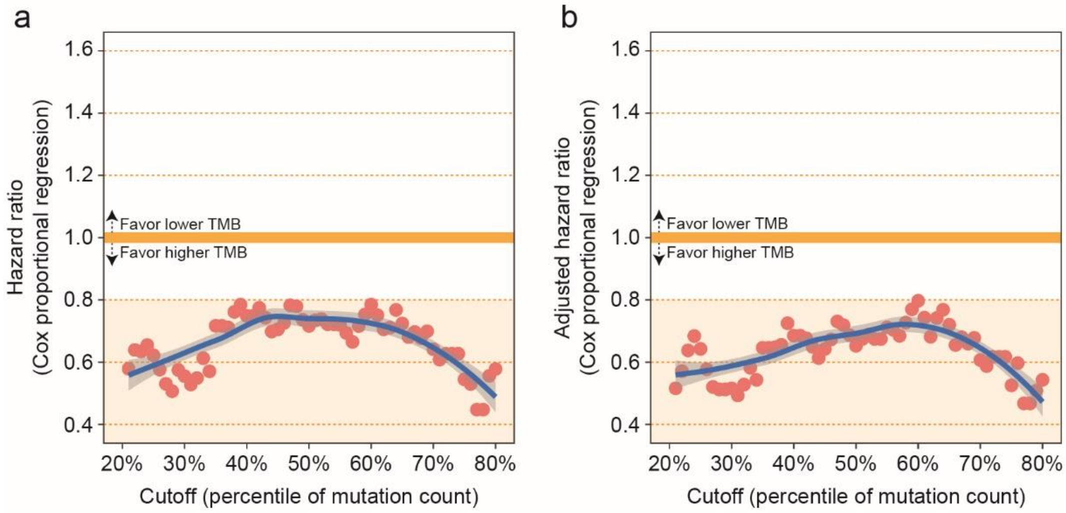

Supplementary Figure 1. Effect of the total mutational burden on the overall survival of patients receiving radiotherapy.

(a) Hazard ratios from the Cox proportional regression of the overall survival were calculated for the irradiated patients. Dots represented the HRs against their corresponding cutoff TMB values in the differing patients into higher and lower TMB groups. The cutoff value for TMB was set to between 20 to 80% and was assessed using a 1% stepwise function. Line with shadow, Loess regression line with a 95% confidence interval. **(b)** The HR were adjusted by gender (male vs female), age (≥ 65yrs vs < 65yrs), and the pathologic diagnosis (lung adenocarcinoma vs squamous cell lung cancer).

Supplementary Figure 2

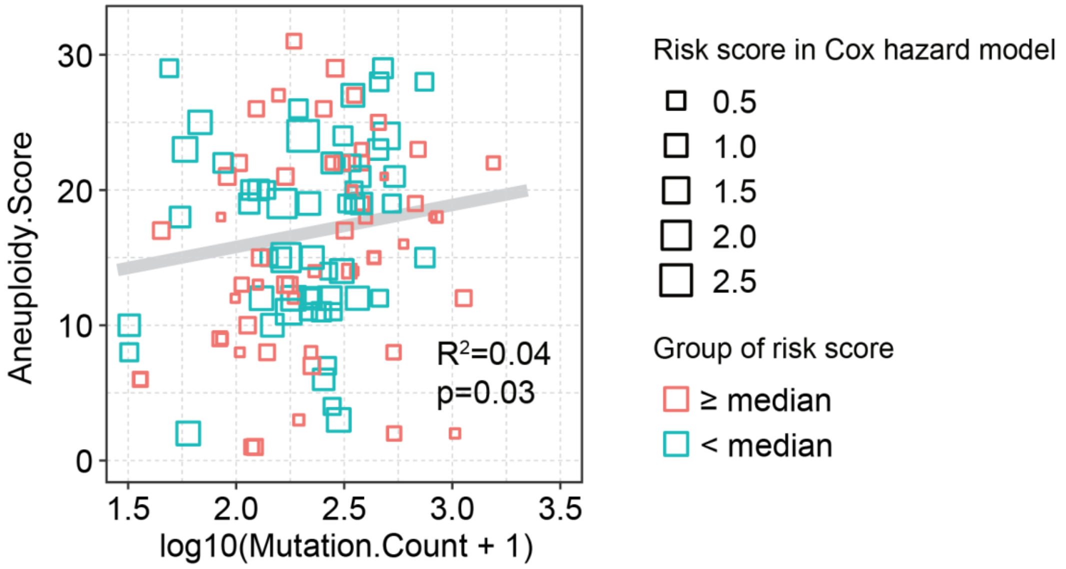

Supplementary Figure 2. Correlation between tumor mutational burden and chromosomal aneuploidy.

The scatterplot shows the correlation between the aneuploidy scores and the mutational loads. TMBs were transformed into log10 scales. The size of square equates to the derived risk score; pink square, patients in higher risk group; green square, patients in lower risk group.

Supplementary Figure 3

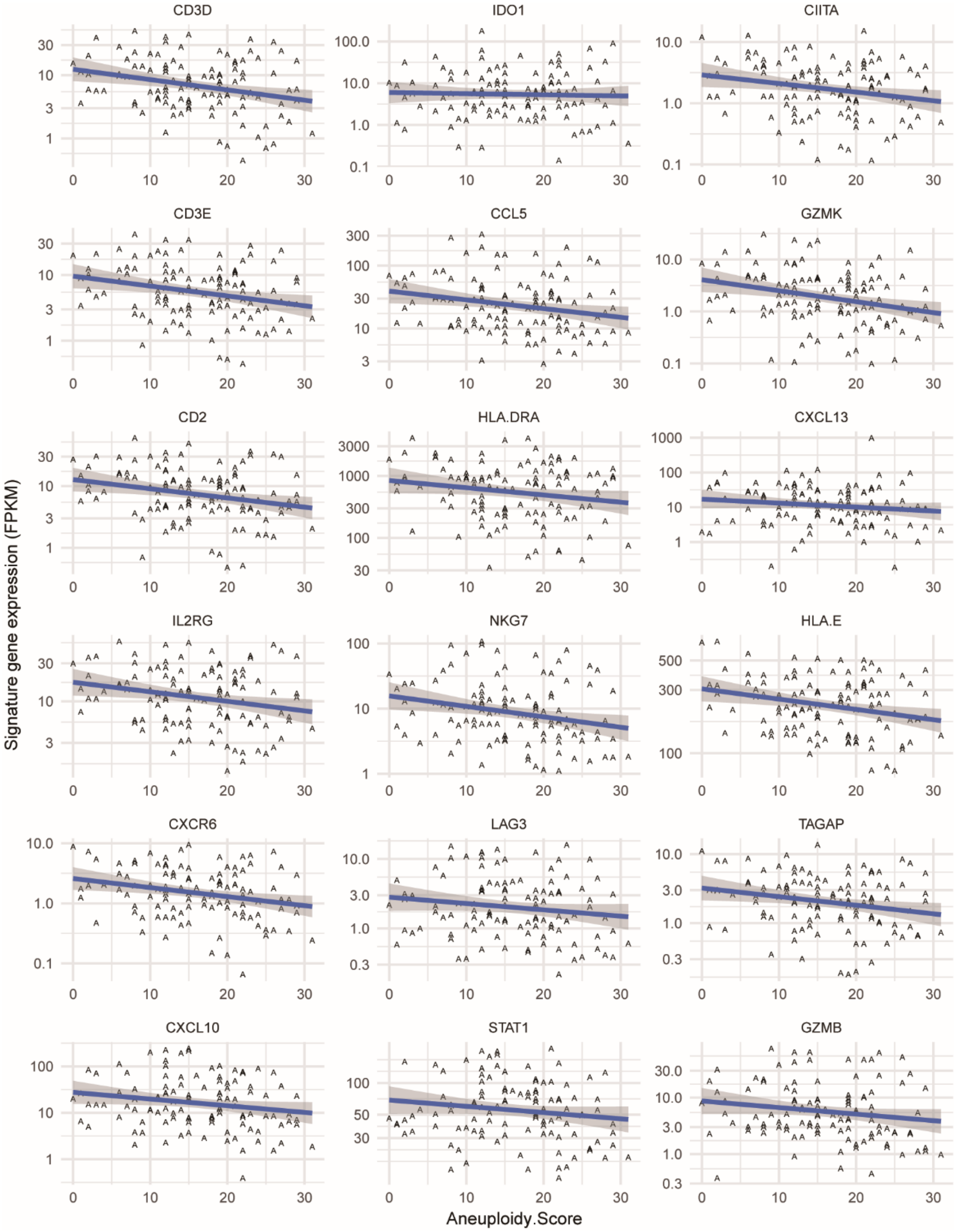

Supplementary Figure 3. Correlation between IFN-γ-related gene expression panel and aneuploidy score.

Scatterplot showed the correlation between aneuploidy score and all 18 genes in IFN-γ-related gene expression panel. Lines with shadows, linear regression with 95% confidence interval. The expression of each gene were transformed in log10 scales. A, aneuploidy score.

Supplementary Figure 4

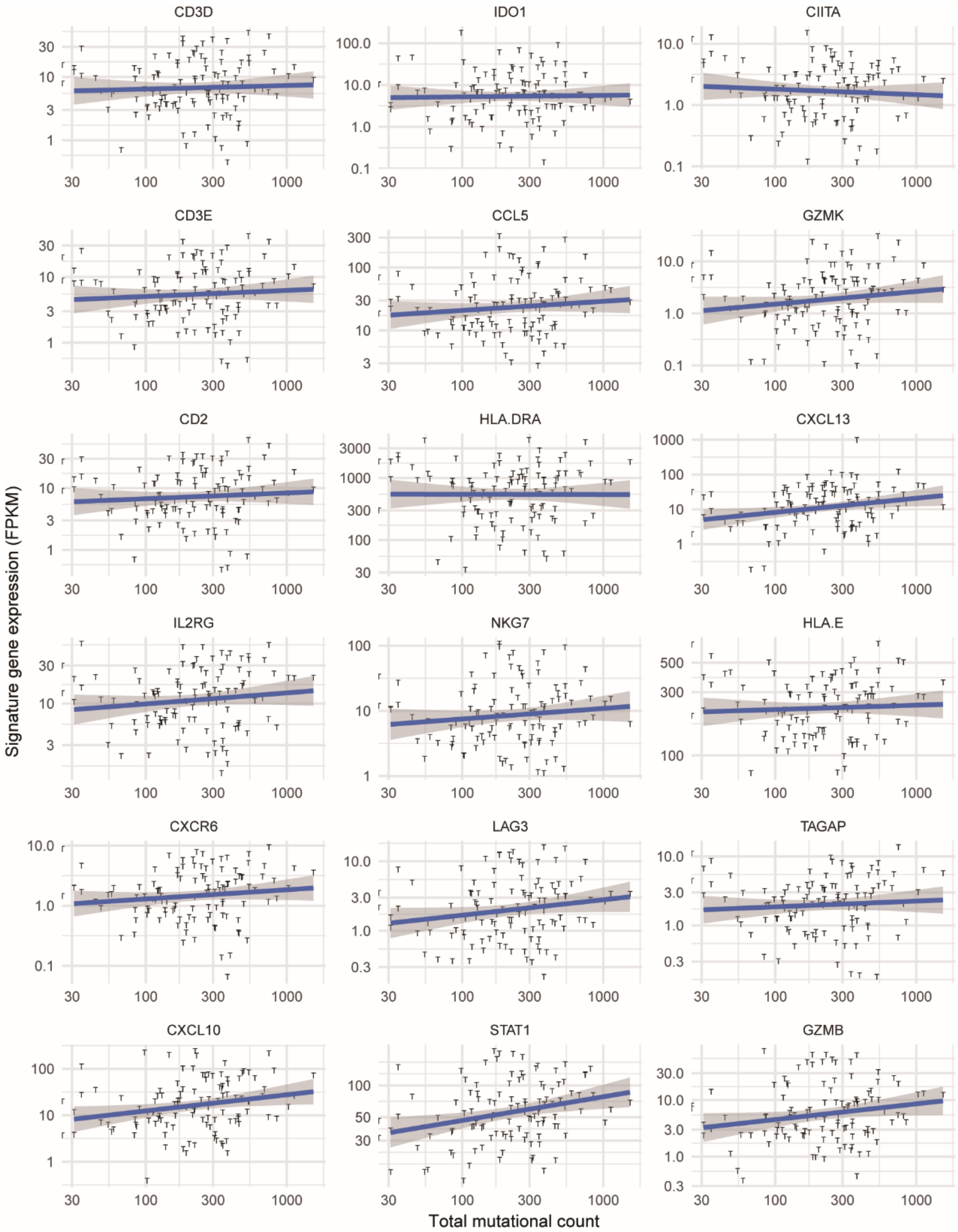

Supplementary Figure 4. Correlation between IFN-γ-related gene expression panel and total mutational burden.

Scatterplot showed the correlation between TMB score and all 18 genes in IFN-γ-related gene expression panel. Lines with shadows, linear regression with 95% confidence interval. The expression of each gene were transformed in log10 scales. T, tumor mutational burden.

Supplementary Figure 5

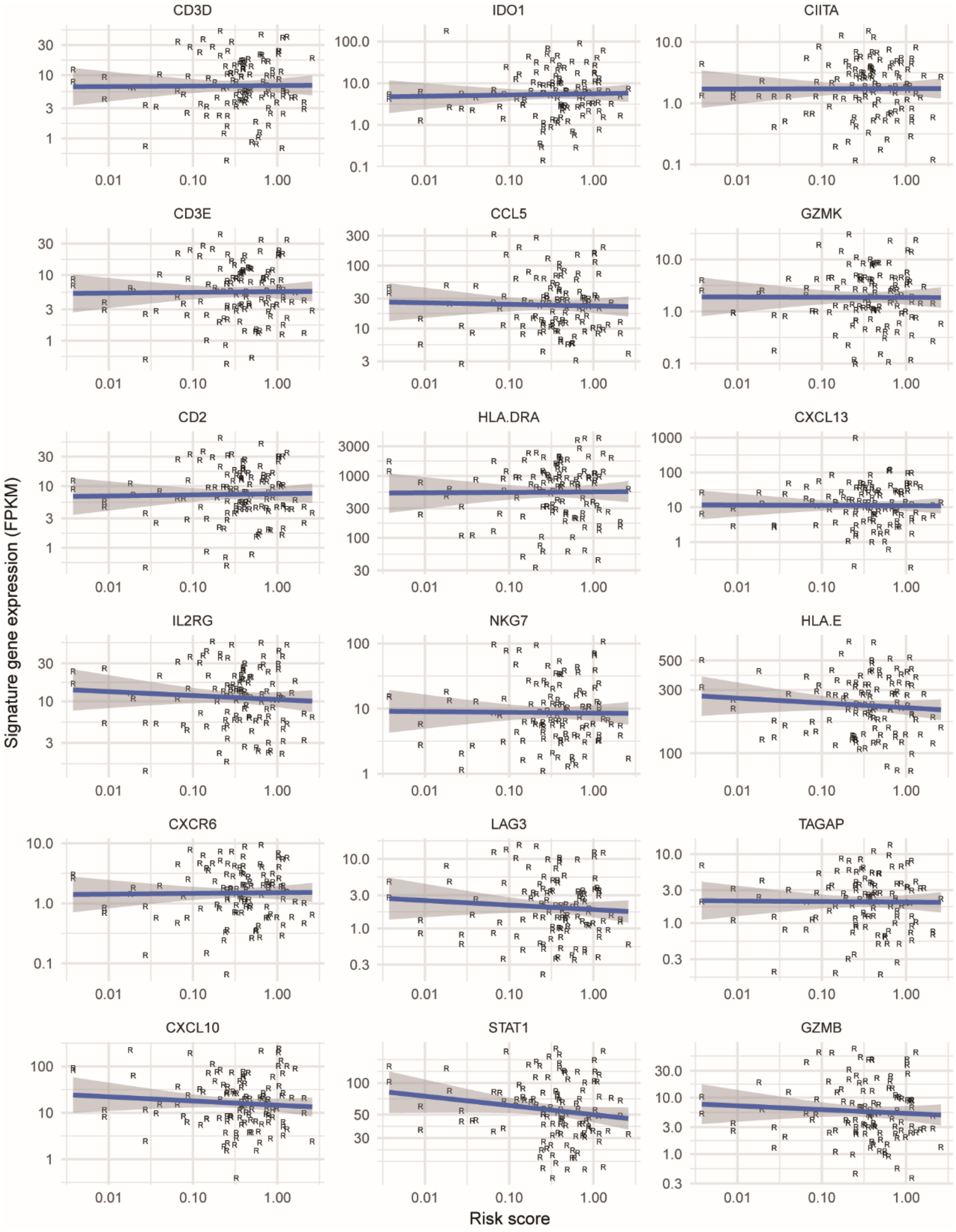

Supplementary Figure 5. Correlation between IFN-γ-related gene expression panel and risk score.

Scatterplot showed the correlation between risk score and all 18 genes in IFN-γ-related gene expression panel. Lines with shadows, linear regression with 95% confidence interval. The expression of each gene was transformed in log10 scales. R, risk score.

Supplementary Figure 6

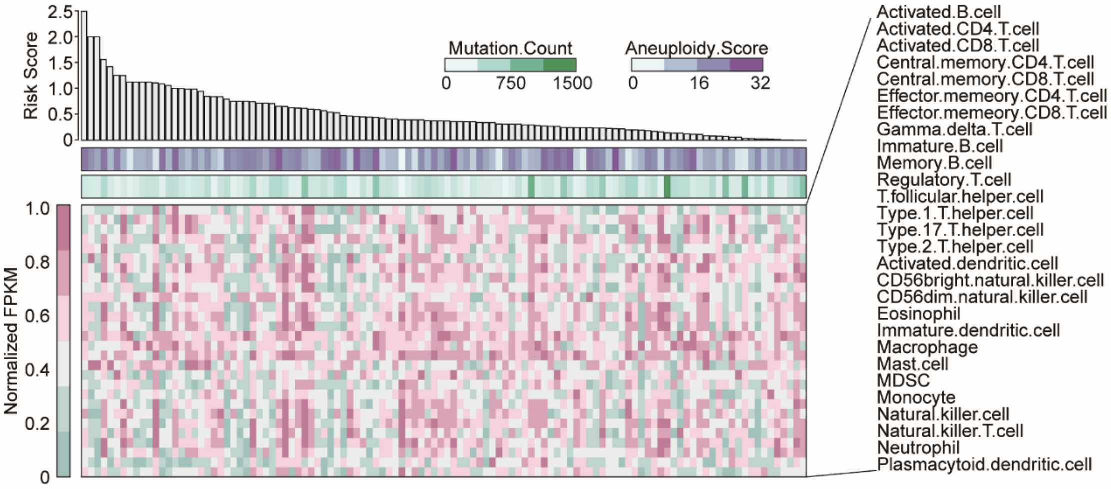

Supplementary Figure 6. Immune cell infiltration for each patient.

The infiltration of 28 types of immune cells for each irradiated patient. The magnitude of immune cell infiltration was estimated by normalized ssGSEA scores. Mutation count, aneuploidy score, as well as risk score were annotated in the upper panel. Patients were ordered by the risk score decreasingly.
