# Supplementary material for: Mutational burden and chromosomal aneuploidy synergistically predict survival from radiotherapy in non-small cell lung cancer
Source: Commun Biol. 2021 Jan 29;4:131. doi: 10.1038/s42003-021-01657-6 (PMC7846582; doi:10.1038/s42003-021-01657-6)
Supplement: Supplementary file 2 — Description of Additional Supplementary Files. [file 42003_2021_1657_MOESM2_ESM.pdf]

Description of Additional Supplementary Files

File name: Supplementary Data 1

Description: Summaries of the discovery cohort

File name: Supplementary Data 2

Description: Multivariate Cox hazard model

File name: Supplementary Data 3

Description: Clinical characteristics between different risk group

File name: Supplementary Data 4

Description: Summaries of the validation cohort

File name: Supplementary Data 5

Description: Clinical characteristics between discovery and validation cohort
