# Supplementary material for: Mutational burden and chromosomal aneuploidy synergistically predict survival from radiotherapy in non-small cell lung cancer
Source: Commun Biol. 2021 Jan 29;4:131. doi: 10.1038/s42003-021-01657-6 (PMC7846582; doi:10.1038/s42003-021-01657-6)
Supplement: Supplementary file 8 — Reporting summary. [file 42003_2021_1657_MOESM8_ESM.pdf]

nature research

Corresponding author(s): YIsong Y. Wan

Last updated by author(s): Sep 20, 2020

Reporting Summary

Nature Research wishes to improve the reproducibility of the work that we publish. This form provides structure for consistency and transparency in reporting. For further information on Nature Research policies, see Authors & Referees and the Editorial Policy Checklist.

Statistics

For all statistical analyses, confirm that the following items are present in the figure legend, table legend, main text, or Methods section.

n/a	Confirmed	
☐	☒	The exact sample size (*n*) for each experimental group/condition, given as a discrete number and unit of measurement
☐	☒	A statement on whether measurements were taken from distinct samples or whether the same sample was measured repeatedly
☐	☒	The statistical test(s) used AND whether they are one- or two-sided<br>*Only common tests should be described solely by name; describe more complex techniques in the Methods section.*
☒	☐	A description of all covariates tested
☒	☐	A description of any assumptions or corrections, such as tests of normality and adjustment for multiple comparisons
☐	☒	A full description of the statistical parameters including central tendency (e.g. means) or other basic estimates (e.g. regression coefficient) AND variation (e.g. standard deviation) or associated estimates of uncertainty (e.g. confidence intervals)
☐	☒	For null hypothesis testing, the test statistic (e.g. *F*, *t*, *r*) with confidence intervals, effect sizes, degrees of freedom and *P* value noted<br>*Give P values as exact values whenever suitable.*
☒	☐	For Bayesian analysis, information on the choice of priors and Markov chain Monte Carlo settings
☒	☐	For hierarchical and complex designs, identification of the appropriate level for tests and full reporting of outcomes
☒	☐	Estimates of effect sizes (e.g. Cohen's *d*, Pearson's *r*), indicating how they were calculated

Our web collection on statistics for biologists contains articles on many of the points above.

Software and code

Policy information about availability of computer code

Data collection	NA
Data analysis	Graph-Pad Software Inc, Version 7.03 and R studio were used for statistical analysis.

For manuscripts utilizing custom algorithms or software that are central to the research but not yet described in published literature, software must be made available to editors/reviewers. We strongly encourage code deposition in a community repository (e.g. GitHub). See the Nature Research guidelines for submitting code & software for further information.

Data

Policy information about availability of data

All manuscripts must include a data availability statement. This statement should provide the following information, where applicable:

- Accession codes, unique identifiers, or web links for publicly available datasets
- A list of figures that have associated raw data
- A description of any restrictions on data availability

For the discovery cohort, 566 patients with lung adenocarcinoma (LUAD, https://www.cbioportal.org/study/summary?id=luad_tcga_pan_can_atlas_2018) and 487 patients with lung squamous cell carcinoma (LUSC, https://www.cbioportal.org/study/summary?id=lusc_tcga_pan_can_atlas_2018) in the TCGA database were enrolled. For 113 patients with “YES” and 738 patients with “NO” in the item “Radiation Therapy” were enrolled as the discovery cohort for patients that had received and not received radiotherapy, respectively. The remaining 202 patients with “NA” for the item “Radiation Therapy” were excluded from subsequent analysis. For the validation cohort, original sequencing data supporting the conclusions of this project have been deposited to the Sequence Read Archive (SRA) under accession number SRP251644.

Field-specific reporting

Please select the one below that is the best fit for your research. If you are not sure, read the appropriate sections before making your selection.

☒ Life sciences ☐ Behavioural & social sciences ☐ Ecological, evolutionary & environmental sciences

For a reference copy of the document with all sections, see nature.com/documents/nr-reporting-summary-flat.pdf

Life sciences study design

All studies must disclose on these points even when the disclosure is negative.

Sample size	For the discovery cohort, as stated in the data availability, all patients with the record of history of radiotherapy were enrolled. For the validation cohort, 34 patients is reasonable sufficient to draw a preliminary validation to our predictive model.
Data exclusions	No data were excluded from the analysis.
Replication	Not applicable.
Randomization	Not applicable.
Blinding	Not applicable.

Reporting for specific materials, systems and methods

We require information from authors about some types of materials, experimental systems and methods used in many studies. Here, indicate whether each material, system or method listed is relevant to your study. If you are not sure if a list item applies to your research, read the appropriate section before selecting a response.

Materials & experimental systems

n/a	Involved in the study	
☒	☐	Antibodies
☒	☐	Eukaryotic cell lines
☒	☐	Palaeontology
☒	☐	Animals and other organisms
☐	☒	Human research participants
☒	☐	Clinical data

Methods

n/a	Involved in the study	
☒	☐	ChIP-seq
☒	☐	Flow cytometry
☒	☐	MRI-based neuroimaging

Human research participants

Policy information about studies involving human research participants

Population characteristics	Age, gender, pathologic type of non-small cell lung cancer, progression-free survival, best of response, TNM stage, intent of treatment and the method of radiotherapy were also record
Recruitment	Patients with the following criteria were enrolled into the validation cohort: 1) diagnosed with NSCLC and received radiotherapy at XinQiao Hospital (Army Medical University, Chongqing), TongJi Hospital (Huazhong University of Science and Technology, Wuhan), or Zhongnan Hospital (Wuhan University, Wuhan); 2) at least 18-years old at diagnosis; 3) availability of radiological surveillance scans; 4) at least 10 slides of 5 um-thick formalin-fixed paraffin-embedded (FFPE) biopsies and peripheral blood mononuclear cell (PBMC) from a total of 2 ml of blood that was obtained at the time of diagnosis.
Ethics oversight	The study was approved by the institutional review board of XinQiao Hospital (Army Medical University, Chongqing), TongJi Hospital (Huazhong University of Science and Technology, Wuhan), and Zhongnan Hospital (Wuhan University, Wuhan). All patients in validation cohort provided written informed consent.

Note that full information on the approval of the study protocol must also be provided in the manuscript.
